# Contrasting genomic consequences of anthropogenic reintroduction and natural recolonization in high‐arctic wild reindeer

**DOI:** 10.1111/eva.13585

**Published:** 2023-08-22

**Authors:** Hamish A. Burnett, Vanessa C. Bieker, Mathilde Le Moullec, Bart Peeters, Jørgen Rosvold, Åshild Ønvik Pedersen, Love Dalén, Leif Egil Loe, Henrik Jensen, Brage B. Hansen, Michael D. Martin

**Affiliations:** ^1^ Centre for Biodiversity Dynamics, Department of Biology Norwegian University of Science and Technology (NTNU) Trondheim Norway; ^2^ Department of Natural History, NTNU University Museum Norwegian University of Science and Technology (NTNU) Trondheim Norway; ^3^ Department of Terrestrial Biodiversity Norwegian Institute for Nature Research (NINA) Trondheim Norway; ^4^ Norwegian Polar Institute Research Department Tromsø Norway; ^5^ Centre for Palaeogenetics Stockholm Sweden; ^6^ Department of Bioinformatics and Genetics Swedish Museum of Natural History Stockholm Sweden; ^7^ Department of Zoology Stockholm University Stockholm Sweden; ^8^ Faculty of Environmental Sciences and Natural Resource Management Norwegian University of Life Sciences Aas Norway; ^9^ Department of Terrestrial Ecology Norwegian Institute for Nature Research (NINA) Trondheim Norway

**Keywords:** conservation genetics, inbreeding, recolonization, reintroduction

## Abstract

Anthropogenic reintroduction can supplement natural recolonization in reestablishing a species' distribution and abundance. However, both reintroductions and recolonizations can give rise to founder effects that reduce genetic diversity and increase inbreeding, potentially causing the accumulation of genetic load and reduced fitness. Most current populations of the endemic high‐arctic Svalbard reindeer (*Rangifer tarandus platyrhynchus*) originate from recent reintroductions or recolonizations following regional extirpations due to past overharvesting. We investigated and compared the genomic consequences of these two paths to reestablishment using whole‐genome shotgun sequencing of 100 Svalbard reindeer across their range. We found little admixture between reintroduced and natural populations. Two reintroduced populations, each founded by 12 individuals around four decades (i.e. 8 reindeer generations) ago, formed two distinct genetic clusters. Compared to the source population, these populations showed only small decreases in genome‐wide heterozygosity and increases in inbreeding and lengths of runs of homozygosity. In contrast, the two naturally recolonized populations without admixture possessed much lower heterozygosity, higher inbreeding and longer runs of homozygosity, possibly caused by serial population founder effects and/or fewer or more genetically related founders than in the reintroduction events. Naturally recolonized populations can thus be more vulnerable to the accumulation of genetic load than reintroduced populations. This suggests that in some organisms even small‐scale reintroduction programs based on genetically diverse source populations can be more effective than natural recolonization in establishing genetically diverse populations. These findings warrant particular attention in the conservation and management of populations and species threatened by habitat fragmentation and loss.

## 1 INTRODUCTION

Species reintroductions are increasingly being used in ecological restoration and biodiversity conservation programmes (Seddon et al., 2014; Taylor et al., 2017; Bubac et al., 2019). Most reintroductions involve translocation of a small number of individuals to establish new populations that may be geographically isolated from the species' current range (Frankham, 2010). Founding populations are often characterized by having a small effective population size, only a subset of the genetic variation that exists in their source populations and limited or no gene flow with other populations (Frankham, 2010; Lynch & Gabriel, 1990). An alternative to reintroduction by translocations is the use of more passive measures that facilitate natural dispersal and recolonization of species' ranges (Scott et al., 2001). Natural recolonization processes may differ from reintroductions in that they require some connectivity with other populations. They can, therefore, be very slow, even in highly mobile species (Hurford et al., 2006; Larter et al., 2000). Especially in fragmented habitats and in species with low dispersal rates, natural recolonization (including recolonization from reintroduced populations) may also involve one or multiple sequential founding events and relative isolation of recolonized populations (Clegg et al., 2002; Pruett & Winker, 2005).

Due to the often small founder population size, both reintroduced and naturally recolonized populations may initially experience strong genetic drift and accumulate inbreeding because individuals are more likely to share common ancestors, which increases homozygosity (Allendorf, 1986; Nei et al., 1975). The levels of genetic diversity in reintroduced and recolonized populations are however also affected by population growth rate, genetic structure and immigration (Biebach & Keller, 2010, 2012; Latch & Rhodes, 2005). For example, inbreeding and genetic drift accumulate over generations at a rate that depends on the population size and the rate of immigration (Whitlock et al., 2000; Willi et al., 2013). Rapid population growth reduces the duration of a population bottleneck and the degree of genetic drift (Allendorf, 1986; Nei et al., 1975). Immigration counteracts the loss of diversity due to drift by introducing unrelated individuals and novel genetic material that replenishes genetic variation and reduces inbreeding rates (Frankham et al., 2017; Latch & Rhodes, 2005; Vucetich & Waite, 2000). The accumulation of inbreeding and genetic drift can allow low‐frequency (partially) recessive deleterious alleles that are rarely homozygous (i.e. masked genetic load [Bertorelle et al., 2022]) to increase in frequency and even become fixed. Consequently, masked genetic load may be converted to realized genetic load (Bertorelle et al., 2022; Wang et al., 1999), which is expected to reduce fitness (i.e. inbreeding depression [Charlesworth & Willis, 2009]). However, this process exposes deleterious variation to selection, potentially purging strongly deleterious recessive alleles and reducing the fitness consequences of future inbreeding (Hedrick & Garcia‐Dorado, 2016; Robinson et al., 2018). Genetic drift also reduces genetic diversity, including potentially adaptive genetic variation that may be important for evolutionary responses necessary to maintain fitness in changing environments (Frankham, 2005; Kardos et al., 2021). Together, these genetic consequences can impact both the short‐ and long‐term viability of populations (Frankham, 2005; Weeks et al., 2011).

Consequently, a key goal in the management of newly reestablished or fragmented populations is to maximize the genetic diversity and minimize drift and inbreeding (Frankham et al., 2017). Several studies have shown that genetic diversity in reintroduced and naturally recolonized populations is often higher in those that receive gene flow from other populations (Biebach & Keller, 2012; Latch & Rhodes, 2005; Malaney et al., 2018), originate from multiple source populations (Huff et al., 2010; Sasmal et al., 2013; Vasiljevic et al., 2022; Williams et al., 2000; Williams & Scribner, 2010) and in reintroductions that use multiple translocations (Cullingham & Moehrenschlager, 2013; Drauch & Rhodes, 2007). Without such mitigating factors, erosion of genetic diversity and accumulation of inbreeding can occur due to isolation and/or slow population growth (Hundertmark & van Daele, 2010; Williams et al., 2002), and may have detrimental population‐level consequences for fitness‐related traits (Wisely et al., 2008) and population growth rates (Bozzuto et al., 2019). Differences in population connectivity and demography between reintroductions and natural recolonizations could, therefore, result in differing genetic consequences for these two paths to population reestablishment. Reintroduced populations may be more isolated from their source than naturally recolonized populations. However, the sequential reestablishment of habitat that often characterizes natural recolonization can result in cumulative founder effects that severely reduce genetic diversity (Clegg et al., 2002; Le Corre & Kremer, 1998). Naturally recolonized populations can thus be more vulnerable to the accumulation of genetic load than reintroduced populations in species or environments with limited dispersal possibilities.

Recent years have seen increased accessibility of genomic data that provide greater power to study population structure, genetic diversity and inbreeding (Supple & Shapiro, 2018), which are important for understanding the genetic outcomes of reintroductions (Hicks et al., 2007; Taylor & Jamieson, 2008; Wright et al., 2014). One such advantage of genomic data is its utility for quantifying inbreeding using runs of homozygosity (RoH). These RoH occur when breeding between individuals that share common ancestors results in offspring with stretches of homozygosity along segments of their homologous chromosomes that both parents inherited from a common ancestor (Kardos et al., 2015, 2016). The ability to quantify the length of RoH segments enables us to distinguish between inbreeding due to recent or more distant shared ancestors of the parents based on the distribution of RoH lengths, giving insights into the demographic history of populations (Brüniche‐Olsen et al., 2018; Druet & Gautier, 2017; Kardos et al., 2017).

While the success of reintroductions has been studied across a variety of taxa including fish (Drauch & Rhodes, 2007), birds (Brekke et al., 2011), insects (White et al., 2017) and ungulates other than reindeer (Grossen et al., 2018), few studies have been able to evaluate and compare their genetic consequences with those from natural population reestablishments. The wild, endemic Svalbard reindeer (*Rangifer tarandus platyrhynchus* Vrolik, 1829) subspecies, with its strong metapopulation structure (Peeters et al., 2020), is a biological system well suited for comparing the genetic consequences of reintroductions to those of natural recolonizations. The number of Svalbard reindeer declined drastically due to overharvesting until 1925, when they were protected, and the subspecies was extirpated from much of the Svalbard archipelago, with evidence of reindeer surviving in four isolated populations totalling ~1000 individuals (Le Moullec et al., 2019; Lønø, 1959). The subspecies has since largely recovered, with natural recolonization and anthropogenic reintroductions restoring most of its former range. Accordingly, Svalbard reindeer are now abundant (~22,000 individuals) (Le Moullec et al., 2019) with most populations relatively stable or increasing in size (Hansen, Pedersen, et al., 2019), and populations previously extirpated are still recovering in number (Le Moullec et al., 2019). Environmental conditions (including sea‐ice coverage [Peeters et al., 2020]) are rapidly changing in the Arctic due to climate change (Isaksen et al., 2022), thus the genetic diversity and genetic structure of reindeer populations may be important for their capacity to adapt to these conditions and influence their future population dynamics. Knowledge of the genomic consequences of reintroductions and natural recolonizations in Svalbard reindeer will, therefore, inform future management of this endemic subspecies, in addition to contributing to a broader understanding of the genetic outcomes of different population reestablishment strategies relevant to the conservation management of other species.

Here, we use whole‐genome sequencing data to investigate the genetic consequences of two Svalbard reindeer reintroductions (each founded by 12 individuals [Aanes et al., 2000; Gjertz, 1995]) and compare these to natural recolonization processes in adjacent, comparable habitats with similar ecological conditions. Specifically, we quantify the degree to which the genetic diversity of the source population was retained after the founder effects and subsequent rapid population growth (Kohler & Aanes, 2004) associated with anthropogenic reintroduction, and whether a signature of this reintroduction could be detected in the form of longer RoH. Additionally, we investigated whether naturally recolonized populations that were not admixed would show different patterns of genetic diversity and inbreeding coefficients compared to reintroduced populations due to the compounding effects of sequential founding events during natural recolonization.

## 2 RESULTS

### 2.1 Sequencing

We sequenced 100 whole genomes of reindeer sampled from a total of 12 populations and sub‐populations, including six sub‐populations originating from two reintroductions (*n* = 46), the reintroduction source population (*n* = 17), two other remnant natural populations (*n* = 13) and three naturally recolonized populations (*n* = 24) across Svalbard. This resulted in a mean nuclear genome sequencing depth of 3.3× for the 90 samples sequenced to a lower target coverage and 23.4× for 10 deep‐sequenced samples, after all filtering (see Figure S1 for distribution of sequencing coverage). Four samples had <0.1× coverage and thus were used only for the site frequency spectrum (SFS) estimates. Genotype likelihoods for 8,255,693 variable sites were calculated from the caribou nuclear genome‐ (Taylor et al., 2019) mapped sequence data after quality filtering. In total, 6,309,215 of these sites remained after removing scaffolds mapping to the bovine X chromosome, and 467,146 sites remained in the dataset used for admixture and PCA analysis after LD pruning. Mean sequencing depth of the mitochondrial genome was >1000×.

### 2.2 Admixture and principal component analyses

**FIGURE 1:**
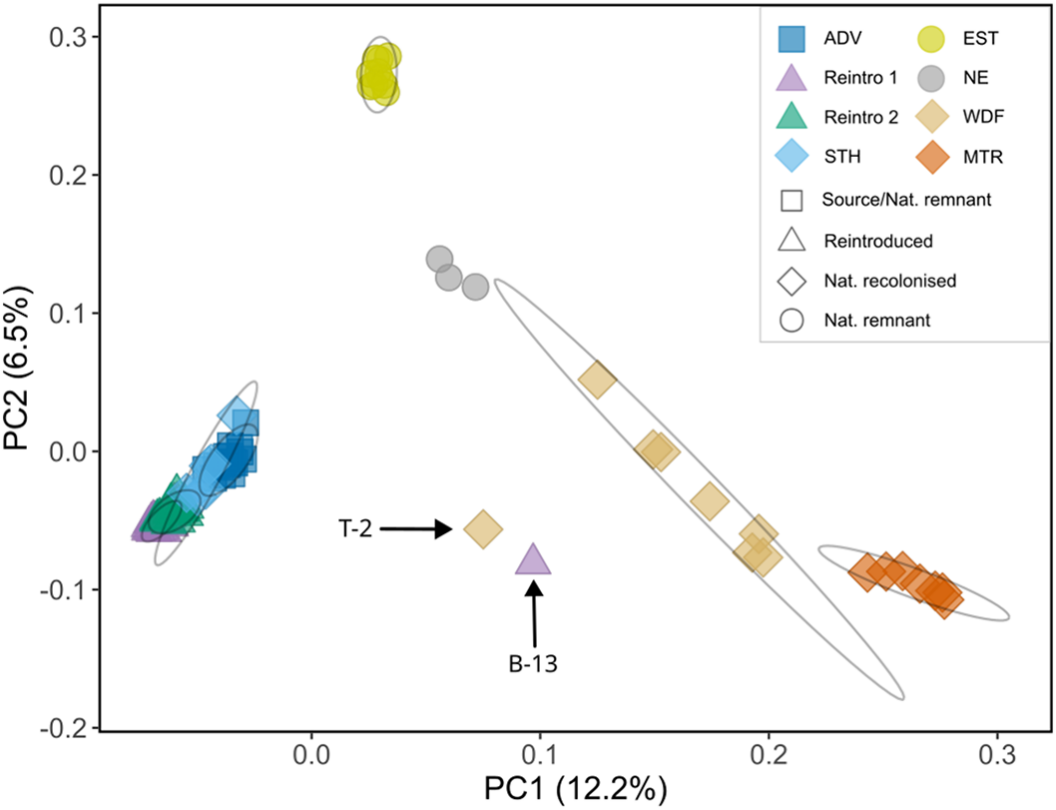
Principal component analysis plot showing PC 1 and 2. Shapes indicate the type of Svalbard reindeer population and colours indicate the sample population. Ellipses represent the 95% CI of the mean PC coordinates for each natural population (except NE due to too few samples) and each reintroduction group. Based on NGSadmix *K* = 2 model results, two individuals (T‐2 and B‐13) that represented admixture between strongly differentiated populations were not included in population ellipse calculations. ADV, Adventdalen; BGR, Brøggerhalvøya; DAU, Daudmannsøyra; EST, Eastern Svalbard; KAF, Kaffiøyra; MTR, Mitrahalvøya; NE, North East Land; NIF, North Isfjorden; PKF, Prins Karls Forland; SAR, Sarsøyra; STH, Southern Spitsbergen; WDF, Wijdefjorden.

**FIGURE 2:**
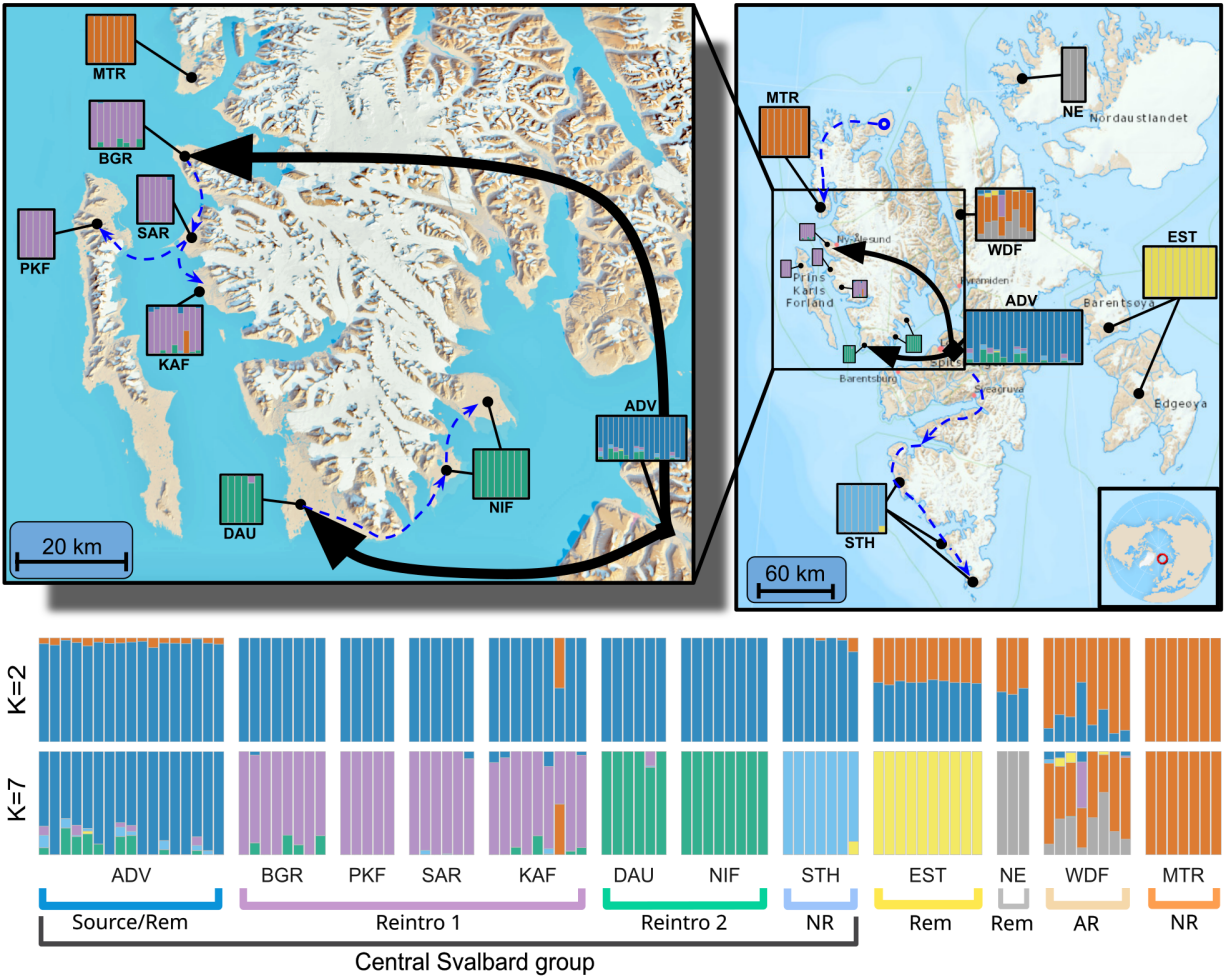
Admixture analysis results from NGSadmix analysis of Svalbard reindeer nuclear genomes. Upper: Admixture proportions for model *K* = 7 (bars), shown at population locations. Arrows indicate translocations for reintroduction 1 and 2; Lower: Admixture proportions for *K* = 2 and *K* = 7 models. Vertical bars represent individual reindeer and colours correspond to genetic cluster assignment. Black arrows indicate reintroduction translocations and dashed blue lines indicate assumed natural recolonization routes. Maps obtained from the Norwegian Polar Institute (toposvalbard.npolar.no). Rem: Natural remnant population; NR: Non‐admixed naturally recolonized population; AR: Admixed naturally recolonized population. ADV, Adventdalen; BGR, Brøggerhalvøya; DAU, Daudmannsøyra; EST, Eastern Svalbard; KAF, Kaffiøyra; MTR, Mitrahalvøya; NE, North East Land; NIF, North Isfjorden; PKF, Prins Karls Forland; SAR, Sarsøyra; STH, Southern Spitsbergen; WDF, Wijdefjorden.

Admixture and principal component analysis (PCA) identified clear genetic structure in the Svalbard reindeer metapopulation (Figures 1 and 2). The optimal number of genetic populations identified using the Δ*K* method was *K* = 2 for the admixture analysis including the whole Svalbard‐wide dataset (Figure S2). On the broadest scale, PCA and the *K* = 2 admixture model show tight clustering among a “central Svalbard” group of populations consisting of the reintroduced, the reintroduction source (ADV), and the naturally recolonized southern Spitsbergen (STH) populations (Figures 1 and 2). Strongly correlated residuals between individuals in the same population in the *K* = 2 model calculated using EvalAdmix (Figure S3) indicate this model was a poor fit to the data and may fail to capture finer scale structure. Admixture analysis only including the Central Svalbard genetic group revealed further genetic substructure with the optimum *K* = 2 (Figure S2). Instead of running additional hierarchical admixture analyses, we examined higher *K*‐value models and the correlation of residuals using EvalAdmix to investigate finer scale genetic structure. We present *K* = 7 in Figure 2 as this reflects the hierarchical level of population structure relevant to the origins of, and admixture between, reintroduced and naturally recolonized populations. This is also the simplest model with a low correlation of residuals (<0.1) within all populations (Figure S3). The *K* = 3–10 and *K* = 5–10 models suggested that the remnant populations in EST and North East Land (NE), respectively, originate from distinct ancestral populations (Figure 2, Figure S4), supported by highly correlated residuals in models that assigned them as admixed populations (Figure S3) and segregation on the first two PC axes. Individuals in the naturally recolonized Wijdefjorden (WDF) population were assigned admixed ancestry from the MTR and NE ancestral populations (models *K* = 5–8), or a distinct ancestral population with some admixture with the ancestral NE population (models *K* = 8–10).

On a finer scale, both PCA (Figures S5 and S6) and admixture analysis (Figure 2, Figure S7) showed clear segregation between Reintroduction 1, Reintroduction 2, ADV and STH, with little admixture between the two reintroductions. Evidence of admixture between reintroduced and natural populations was found in only one individual “B‐13” from KAF (Reintroduction 1) that carried approximately 50% MTR ancestry, and one individual in WDF “T‐2” that carried approximately 50% Reintroduction 1 ancestry (Figures 1, 2 and Figure S4).

### 2.3 *F*_ST_ analysis

**FIGURE 3:**
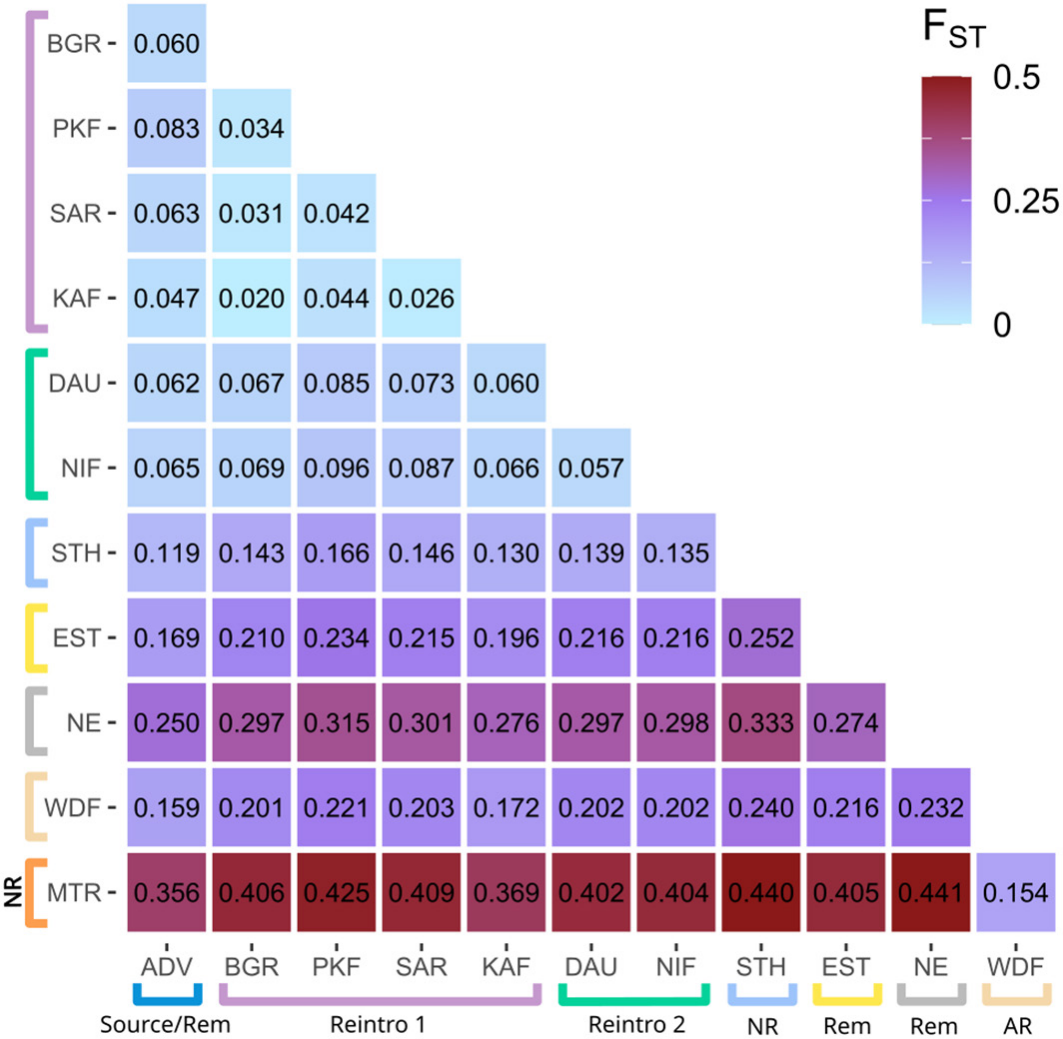
Pairwise *F*_ST_ heatmap for each Svalbard reindeer population based on folded SFS. Coloured brackets correspond to admixture groups assigned by the *K* = 7 model. Rem: Natural remnant population; NR: Non‐admixed naturally recolonized population; AR: Admixed naturally recolonized population. ADV, Adventdalen; BGR, Brøggerhalvøya; DAU, Daudmannsøyra; EST, Eastern Svalbard; KAF, Kaffiøyra; MTR, Mitrahalvøya; NE, North East Land; NIF, North Isfjorden; PKF, Prins Karls Forland; SAR, Sarsøyra; STH, Southern Spitsbergen; WDF, Wijdefjorden.

Pairwise *F*_ST_ estimates showed very strong genetic structure, and largely supported admixture and PCA results (Figure 3). Populations assigned in admixture analyses to the same reintroduction showed lower pairwise *F*_ST_ values between each other than populations assigned to the other reintroduction. Similar levels of genetic differentiation were found in comparisons between the source population and both groups of reintroduced populations, and between the two groups of reintroduced populations. The naturally recolonized population at MTR was clearly the most genetically distinct, showing extremely high differentiation (>0.35) to all other populations except Widefjorden.

#### 2.3.1 Mitochondrial haplotype diversity

**FIGURE 4:**
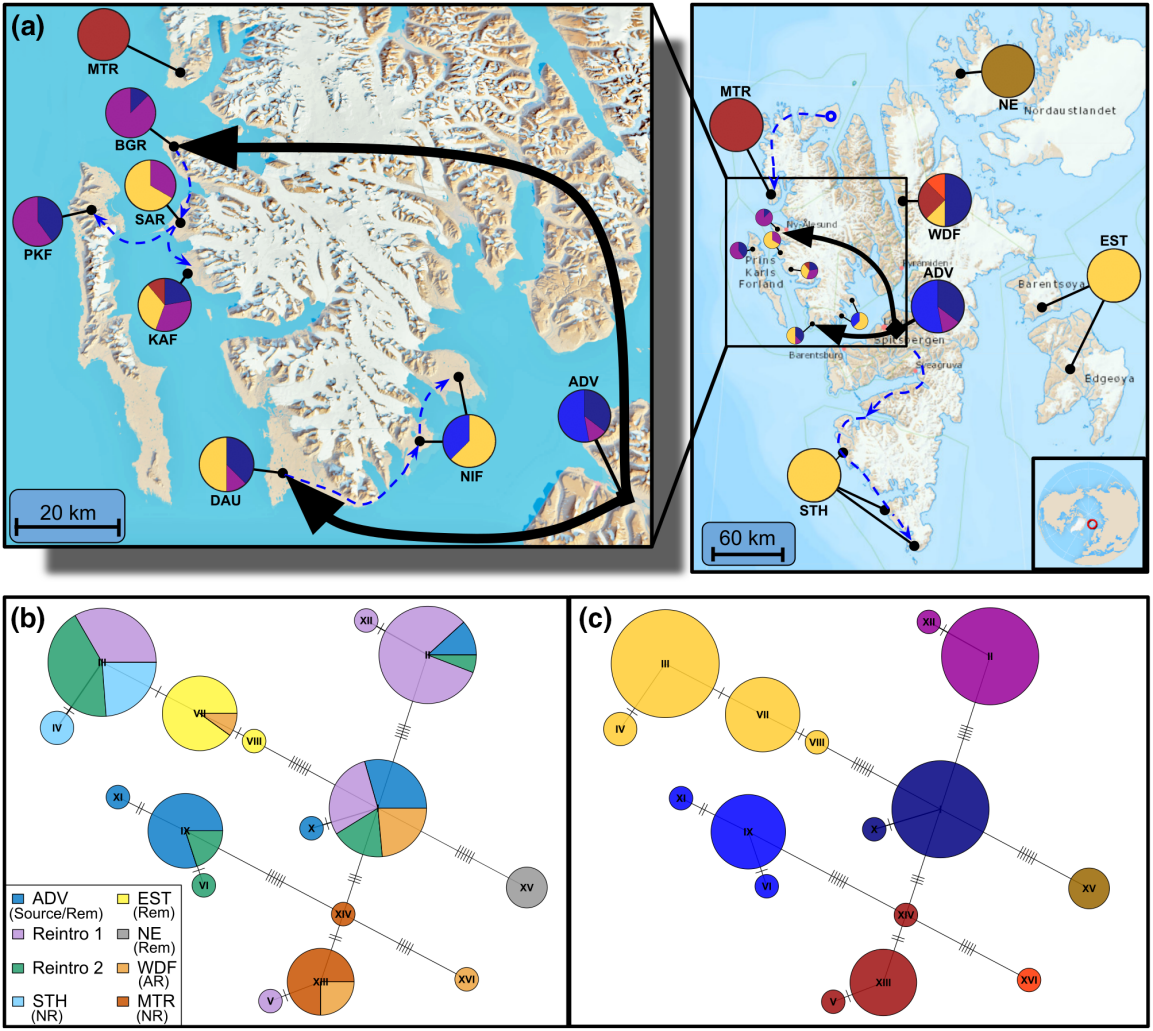
Svalbard reindeer mtDNA haplotype analysis. (a) Population haplogroup composition. Colours indicate haplogroups shown in panel c. Black arrows indicate reintroduction translocations and dashed blue lines indicate assumed natural recolonization routes; (b) Median‐joining mitochondrial haplotype network constructed using an uncorrected number of nucleotide differences (colours represent populations); (c) Grouping of haplotypes into haplogroups connected by links with less than 3 nucleotide differences. Maps obtained from the Norwegian Polar Institute (toposvalbard.npolar.no). Rem: Natural remnant population; NR: Non‐admixed naturally recolonized population; AR: Admixed naturally recolonized population. ADV, Adventdalen; BGR, Brøggerhalvøya; DAU, Daudmannsøyra; EST, Eastern Svalbard; KAF, Kaffiøyra; MTR, Mitrahalvøya; NE, North East Land; NIF, North Isfjorden; PKF, Prins Karls Forland; SAR, Sarsøyra; STH, Southern Spitsbergen; WDF, Wijdefjorden.

We detected 38 variant sites among the 96 full mtDNA genomes, comprising 16 unique haplotypes (Figure 4, Table S1). Haplotypes could be grouped into seven distinct haplogroups with a maximum of two substitutions separating each haplotype from its nearest neighbour within the haplogroup (Figure 4c). Mitochondrial DNA haplotype diversity showed a similar pattern to nuclear genetic analyses. Haplotypes in MTR, NE and EST were not found in any other population except WDF, which carried a mixture of haplotypes found in every population except STH and NE, plus one highly unique haplotype (Figure 4a). Overall, populations with central Svalbard ancestry (ADV, STH and reintroductions) shared similar haplogroups, with the notable exception that the most common haplogroup among reintroduced populations and STH was not found in the ADV source population but instead in EST. Within this shared haplogroup, the haplotypes found in STH and the reintroduced populations were mutually exclusive to those in EST, but differed by as little as a single mutation (Figure 4b). The populations KAF and NIF, founded by natural dispersal from Reintroduction 1 and 2 respectively, carried haplotypes belonging to haplogroups not found in other reintroduced populations (Figure 4a). The KAF individual admixed with MTR (Figure 2) carried a unique haplotype from the MTR haplogroup, and almost half of the NIF samples carried haplotypes in a haplogroup otherwise found only in ADV (Figure 4a).

Haplotype richness was strongly correlated to mean population genome‐wide heterozygosity (Pearson correlation *r* = 0.86, *p* = 0.013). Each of the two reintroduction groups (populations combined) had similar haplotype richness to ADV, and higher than all other natural populations except WDF (Table S1 and Figure S8).

#### 2.3.2 Heterozygosity analysis and coalescent Ne

**FIGURE 5:**
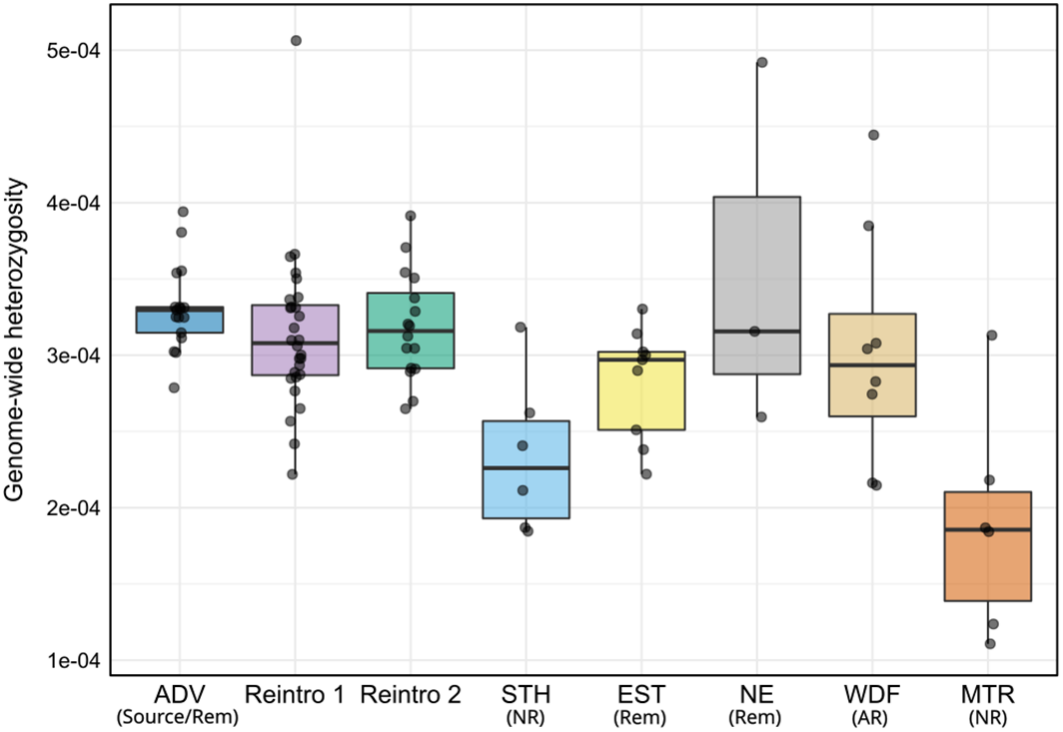
Svalbard reindeer genome‐wide heterozygosity estimates using sequence data downsampled to 2.5× coverage. Reintroduced populations were grouped into Reintroductions 1 and 2 based on the admixture analyses. Rem: Natural remnant population; NR: Non‐admixed naturally recolonized population; AR: Admixed naturally recolonized population. ADV, Adventdalen; BGR, Brøggerhalvøya; DAU, Daudmannsøyra; EST, Eastern Svalbard; KAF, Kaffiøyra; MTR, Mitrahalvøya; NE, North East Land; NIF, North Isfjorden; PKF, Prins Karls Forland; SAR, Sarsøyra; STH, Southern Spitsbergen; WDF, Wijdefjorden.

Heterozygosity was estimated for each sample individually. The median genome‐wide heterozygosity for all individuals in this study with coverage >2.5× was 3.02 × 10^−4^. Populations originating from the first and second reintroductions had slightly lower median genome‐wide heterozygosities (3.08 × 10^−4^, IQR 4.59 × 10^−5^ and 3.16 × 10^−4^, IQR 4.94 × 10^−5^ respectively) than the ADV source population (genome‐wide heterozygosity 3.30 × 10^−4^, IQR 1.68 × 10^−5^, Figure 5), but overall, there was weak evidence that heterozygosity was different between reintroduced individuals (both populations combined as they have almost identical mean heterozygosity and IQR values) and those from ADV (Mann–Whitney *U* test, *W* = 483, *p* = 0.081). In contrast to the remnant natural and admixed recolonized populations with intermediate heterozygosities (EST, NE and WDF), the two non‐admixed naturally recolonized populations MTR (median 1.86 × 10^−4^, IQR 7.14 × 10^−5^) and STH (median 2.26 × 10^−4^, IQR 6.37 × 10^−5^) had very low heterozygosity. These recolonized populations had markedly lower heterozygosity than the reintroduced populations combined (*W* = 240, *p* < 0.001 and *W* = 235, *p* < 0.001, respectively). Coalescent effective population size (*N*_e_) showed similar patterns to heterozygosity (Table S2), with median heterozygosity and *N*_e_ strongly correlated (Pearson correlation *r* = 0.79, *p* = 0.019). ADV and WDF showed the highest *N*_e_ (5744 and 5832, respectively), while MTR had the lowest (4220). Reintroduction 1 had a lower *N*_e_ (5042) than Reintroduction 2 (5544) and STH (5170), while EST and NE had similar intermediate *N*_e_ estimates (5366 and 5310, respectively).

#### 2.3.3 Inbreeding

**FIGURE 6:**
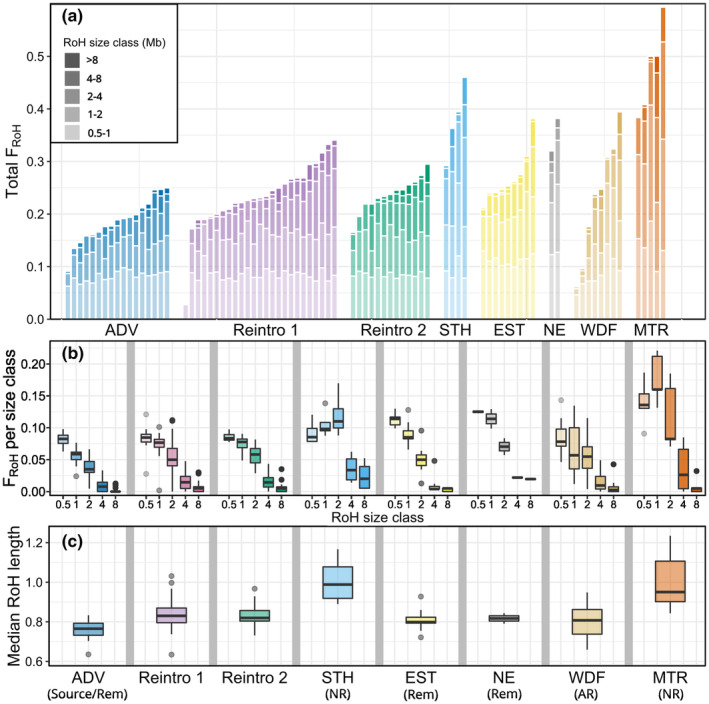
(a) Cumulative total *F*_ROH_ from the five Runs of Homozygosity (RoH) size classes (0.5–1 Mbp, 1–2 Mbp, 2–4 Mbp, 4–8 Mbp and >8 Mbp) with each bar representing an individual Svalbard reindeer genome; (b) Proportion of individual genomes within RoH of each size classes; (c) Median RoH lengths of individuals in each population. Rem: Natural remnant population; NR: Non‐admixed naturally recolonized population; AR: Admixed naturally recolonized population. ADV, Adventdalen; BGR, Brøggerhalvøya; DAU, Daudmannsøyra; EST, Eastern Svalbard; KAF, Kaffiøyra; MTR, Mitrahalvøya; NE, North East Land; NIF, North Isfjorden; PKF, Prins Karls Forland; SAR, Sarsøyra; STH, Southern Spitsbergen; WDF, Wijdefjorden.

We detected 21,990 RoH longer than 0.5 Mbp across the 83 genomes included in the analysis, ranging from 0.5 Mbp to 12.76 Mbp in length and covering between 3% and 59% of individuals' genomes (Figure 6). Both reintroduced populations showed a higher mean inbreeding coefficient (Reintroduction 1 *F*_ROH_ 0.236 ± 0.063 SD, Reintroduction 2 *F*_ROH_ 0.237 ± 0.033) relative to their source population in Adventdalen (*F*_ROH_ 0.186 ± 0.042). The higher inbreeding in reintroduced populations was due to greater coverage of moderate length RoH (between 1 and 8 Mbp) that resulted in increased median RoH lengths (Figure 6), indicating a reduced effective population size in the more recent past compared to the ADV source population.

Non‐admixed naturally recolonized populations MTR and STH had the highest inbreeding coefficients (mean *F*_ROH_ 0.477 ± 0.084 and 0.377 ± 0.07, respectively) and longest median RoH lengths (Figure 6). We found the strongest signals of recent inbreeding in STH, with a high proportion of individuals' genomes covered by RoH >4 Mbp relative to other populations and a peak in *F*_ROH_ in the 2–4 Mbp class (Figure 6b). In MTR, *F*_ROH_ was highest in the 1–2 Mbp class with a large contribution from all other size classes <8 Mbp relative to other populations, but very long (>8 Mbp) RoH were rare. The admixed naturally recolonized population in WDF had the highest variation in total *F*_ROH_ consistent with high variation in the proportion of individuals' shared ancestry. The two remnant natural populations EST and NE both had high *F*_ROH_ in short RoH classes compared to the ADV population, with EST having low *F*_ROH_ in intermediate and long size classes comparable to ADV and the two samples from NE showing relatively high *F*_ROH_ in all size classes.

## 3 DISCUSSION

By analysing whole genome sequences from 100 Svalbard reindeer across their range, we have quantified important genomic consequences and contrasts of population recovery through anthropogenic reintroductions versus natural recolonizations in the previously severely overharvested subspecies. We found strong archipelago‐wide genetic structure, including two distinct genetic clusters corresponding to two reintroductions from a common source, with little evidence for extensive admixture between reintroduced and sampled natural populations (Figures 1, 2, 3, 4). Our results show that reintroduced populations also maintained comparative levels of heterozygosity to their source population (Figure 5), although founder effects resulted in a small increase in the length and coverage of RoH across the genomes of reintroduced individuals (Figure 6). This contrasted strongly with non‐admixed naturally recolonized populations, which had markedly lower genetic diversity and a greater proportion of their genomes comprising RoH. This suggests that non‐admixed, naturally recolonized populations may be more vulnerable to the accumulation of genetic load and loss of adaptive variation than reintroduced populations, even when the latter originate from just a handful of individuals.

### 3.1 Effect of reintroduction versus recolonization on genome‐wide diversity

We estimated genome‐wide heterozygosity and analysed the distribution of RoH lengths to separate the contribution of ancient and more recent demographic history, associated with reintroduction and recolonization, to patterns of genome‐wide variation. Our analyses identified a weak signal of founder effects in both reintroductions, which had higher total inbreeding coefficients and longer RoH, but no significant reduction in average heterozygosity compared to the source population. The increased inbreeding in reintroduced populations was also accompanied by lower coalescent *N*_e_ estimates, with Reintroduction 1 showing a greater reduction (12%) than Reintroduction 2 (4%) compared to the source population. Similar genomic signatures of reintroductions have previously been identified, including in European bison *Bison bonasus* (Druet et al., 2020), Magpie‐robins *Copsychus sechellarum* (Cavill et al., 2022) and ibex *Capra ibex* (Grossen et al., 2018). The increased inbreeding in reintroduced populations was attributable to a greater proportion of the genome in RoH >1 Mbp, including in the 1–2 and 2–4 Mbp range. These size classes reflect shared ancestry and are thus indicative of effective population size approximately 25–50 and 12–25 generations ago given a recombination rate of ~1 cM/Mbp (Kardos et al., 2017; Thompson, 2013), i.e. before the time of the reintroduction translocation, given an average generation time of 5–6 years. We found only a small increase in the coverage of long RoH (4–8 Mbp), which is the expected length of RoH caused by shared ancestors 6–12 generations ago (around the time of the reintroduction), indicating our analysis may have underestimated RoH lengths. However, generation time may have been reduced in the post‐reintroduction period of strong population growth due to an abundance of resources resulting in an earlier age of first reproduction and high fecundity (Giaimo & Traulsen, 2023). Nevertheless, a low frequency of long RoH relative to naturally recolonized populations suggests lower levels of recent inbreeding among reintroduced individuals. The founding population sizes of 12 Svalbard reindeer have thus been sufficient to maintain most of the heterozygosity of the source population and avoid serious accumulation of inbreeding in both reintroductions, both of which are a key concern for reintroduced populations (Frankham et al., 2017; Weeks et al., 2011).

We found fewer long RoH in reintroduced Svalbard reindeer than reported for alpine ibex (Grossen et al., 2018) and European bison (Druet et al., 2020), and our results indicate that, like the source population (ADV), short RoH (i.e. inbreeding due to ancient demography) still contribute the most to the total inbreeding coefficients of reintroduced individuals. Rapid population growth immediately after reintroduction (Aanes et al., 2000) and the extensive overlap between generations in reindeer are both characteristics that reduce the loss of genetic diversity after population bottlenecks (Allendorf, 1986; Nei et al., 1975). Furthermore, past and possibly ongoing dispersal among the secondary sub‐populations recolonized from the initial reintroduced populations (Hansen et al., 2010; Stien et al., 2010) may have buffered against the effects of sequential founder events. Similar outcomes have been observed in reintroductions of a range of vertebrate and invertebrate species where rapid population growth occurred after reintroduction (Brekke et al., 2011; Hicks et al., 2007; Murphy et al., 2015; White et al., 2017). In particular, populations of other ungulates colonized by only a few founding individuals have been shown to retain high levels of heterozygosity as a result of overlapping generations and rapid population expansion (Kaeuffer et al., 2007; Kekkonen et al., 2012). In contrast, populations reintroduced with few founders that remained small for several generations have shown a pronounced reduction in genetic diversity (Williams et al., 2002; Wisely et al., 2008).

In contrast to the reintroductions, the natural recolonization of STH and MTR resulted in populations with very low nuclear genome‐wide heterozygosity, mtDNA haplotype diversity and high total inbreeding coefficients with a longer distribution of RoH. In STH, high *F*_RoH_ across the 1–2, 2–4 and 4–8‐Mbp size classes, with a peak in the 2–4‐Mbp range, indicates small *N*_e_ around the same time as the reintroductions (i.e. during the recolonization period) (Figure 6b). STH also has the highest proportion of genomes within RoH >4 Mbp, indicating smaller *N*_e_ in recent generations that may be due to founder effects associated with recolonization more recently than in the reintroduced populations. This evidence of smaller recent *N*_e_ in STH than in Reintroduction 1 contrasts with comparable coalescent *N*_e_ estimates between the two populations. This could reflect a similar ancestral *N*_e_, but it also could be that our estimate of coalescent *N*_e_ in STH is inflated compared to individual‐level diversity due to population structure within the STH samples, which were collected from a larger and more fragmented geographic area than other populations. Long RoH may be particularly significant to conservation as they represent younger haplotypes that have been exposed to less selection than older haplotypes, making them more likely to harbour deleterious mutations (Bortoluzzi et al., 2020) and have a greater impact on fitness (Stoffel et al., 2021; Szpiech et al., 2013). The very high frequency of short RoH in the MTR genomes and a low coalescent *N*_e_ estimate suggests this population originates from a source with a historically small population size (i.e. prior to recolonization), and high coverage of moderate length RoH indicates founder effects or small *N*_e_ during the last 25 generations (i.e. during recolonization). ADV, the two reintroduced populations and the naturally recolonized STH all had a similar average proportion of genomes within RoH <1 Mbp, indicating similar demographic histories >50 generations ago, consistent with these populations sharing a common origin as indicated by our admixture results. Therefore, differences in patterns of genome‐wide diversity in STH and the reintroduced populations likely reflect differences between anthropogenic reintroductions and natural recolonization, rather than differences in genetic diversity between their ancestral populations. The extremely low levels of heterozygosity and high inbreeding levels in MTR are thus likely a result of a source population with a historically small population size that harboured little genetic diversity prior to recolonization, in addition to more recent founder effects associated with the recolonization process. Indeed, this is consistent with the reported population size of reindeer in the isolated North‐West Spitsbergen area being only 2–300 individuals, 30 years after the historical overharvesting was ended (Lønø, 1959).

Regional‐scale population size estimates show comparable population sizes and strong population growth in both reintroduced and naturally recolonized areas (Table S2; Le Moullec et al., 2019) with comparable habitat quality (Pedersen et al., 2023). The decreased heterozygosity and increased frequency of longer RoH in naturally recolonized compared to reintroduced populations is, therefore, not likely due to differing population sizes and growth rates resulting from environmental differences. Instead, this likely reflects multiple founder effects from a sequential recolonization process (Peeters et al., 2020), potentially involving few dispersing individuals, which was not a characteristic of the anthropogenic reintroduction. Sequential dispersal and establishment of isolated peninsulas and valleys during the recolonization of STH and MTR may have caused cumulative effects from multiple founder events, reducing *N*_e_ and eroding genetic diversity (Clegg et al., 2002; Le Corre & Kremer, 1998; Pruett & Winker, 2005), resulting in increased inbreeding and long RoH despite having comparable current regional population sizes to reintroduced populations.

Our inferences regarding the timing of past demography based on RoH length distributions should be considered only as relative between populations and may not accurately reflect the demographic history in an absolute number of generations. Inferences of demographic history using RoH length distributions are imprecise because spatial and temporal variation in generation times and the random nature of recombination result in high variation around the mean expected length of RoH (Druet & Gautier, 2017). Moreover, sequencing coverage, sequencing error rates, biased genotype likelihood estimates, as well as filtering and parameter settings can all affect estimates of heterozygosity (de Jager et al., 2021; Fuentes‐Pardo & Ruzzante, 2017; Sánchez‐Barreiro et al., 2021), and thus RoH length and frequency (Duntsch et al., 2021). We downsampled sequence data to allow unbiased comparison between samples and populations with varying levels of coverage, and to maximize the sample size for both the genome‐wide heterozygosity and RoH analyses. This may limit the direct comparison of our estimates of heterozygosity to those from other studies using higher‐coverage sequence data.

RoH analysis using low coverage sequence data is also sensitive to filtering, and may falsely identify multiple short RoH as a single longer RoH, or miss RoH altogether (Duntsch et al., 2021). However, the shorter‐than‐expected distribution of RoH in reintroduced individuals, given the known timing of the reintroduction founder effects, suggests our analysis underestimated RoH lengths, and this is consistent with an excess of short (<50‐Kbp) gaps between RoH in our data, breaking up otherwize long RoH (Figure S9). Several factors could break up true RoH and contribute to downwardly biased RoH length distributions: (1) Sequencing errors resulting in false heterozygosity can break up RoH, and will have a larger effect on accurate identification of longer RoH (MacLeod et al., 2013); (2) Any RoH spanning the genome assembly scaffold edges will be broken up (Brüniche‐Olsen et al., 2018), thus lacking a chromosome‐level genome assembly, we only included scaffolds longer than 10 Mbp in RoH analyses; (3) Errors in mapping sequence reads or structural variation between the caribou reference genome assembly and the Svalbard reindeer genome could also break up long RoH. Reanalysing RoH after allowing one <50‐Kbp gap within each 1‐Mbp segment (similar to Wilder et al., 2022) gave qualitatively similar results but showed more long RoH which aligned more closely with the known timing of the reintroduction founder events (Figure S10).

### 3.2 Genetic structure within the Svalbard reindeer metapopulation

Admixture, *F*_ST_ and mitochondrial haplotype analyses identified strong genetic structure across the archipelago, in some cases even over short geographical distances, confirming patterns identified with microsatellite data in Peeters et al. (2020). Such genetic structure is typical of ungulate populations with a history of population fragmentation and bottlenecks due to past harvesting pressure (Haanes et al., 2010; Williams et al., 2002). On a finer scale, this study reveals population structure within the Central Svalbard group, that is, between the source and reintroduced populations, and among reintroduced populations. The two distinct genetic clusters among reintroduced populations corresponded to the two separate reintroductions to isolated peninsulas on the west coast of Spitsbergen. Pairwise *F*_ST_ estimates reveal both reintroductions have resulted in a similar degree of genetic divergence from the source population. Founder effects and subsequent genetic drift commonly induce structure between reintroduced populations and their sources, typically reflecting isolation from the source population (Andersen et al., 2014; Brekke et al., 2011; Grossen et al., 2018; Latch & Rhodes, 2005; Williams et al., 2002).

Close genetic clustering of multiple sub‐populations colonized from a common reintroduced founder population is characteristic of populations manipulated by reintroduction programmes (Andersen et al., 2014; Grossen et al., 2018). We found only weak genetic structure among populations originating from the first reintroduction, except for the rather isolated island PKF, which population showed little admixture with other reintroduced populations (Figure S7), reflecting low dispersal across the sea (Peeters et al., 2020). Population monitoring after the reintroduction to BGR (Reintroduction 1) recorded substantial movement between BGR, SAR and KAF (Hansen et al., 2010; Stien et al., 2010), but GPS collar data from ~200 reindeer‐years and resighting data from ~300 marked reindeer suggest that such exchange of individuals is now rare, with no more than five observations in the past decade (Hansen et al., unpubl. data). This is consistent with the observed lack of fjord ice in recent decades.

Our results indicate little gene flow between reintroduced populations and sampled natural populations. Only one individual from a reintroduced population was identified as admixed with a natural population, with admixture proportions consistent with an F1 offspring resulting from a mating between individuals in Reintroduction 1 and MTR genetic clusters. This individual carried a unique haplotype that differs by only a single mutation from MTR haplotypes, suggesting female dispersal from the north. MTR and BGR, the closest population sampled in Reintroduction 1, are separated by only 15 km across the mouths of Kongsfjorden and Krossfjorden, a span of water which has rarely or never frozen over since the reintroduction (Pavlova et al., 2019; Urbański & Litwicka, 2022). This lack of sea ice as a movement corridor, in combination with tide‐water glaciers and steep mountains inhibiting alternative dispersal routes, has likely prevented gene flow and contributed to the extreme degree of genetic differentiation between these geographically proximate populations. On the contrary, it is likely that these populations were more closely related in the past, that is, before the local extirpations due to overharvest and the subsequent reintroduction from central Spitsbergen (to BGR) and recolonization from the North (to MTR). This illustrates how both reintroductions and recolonization may cause dramatic changes in population‐genetic structuring and diversity.

An exception of the clearly separated reintroduced versus naturally recolonized populations occurred along the northern side of Isfjorden in central Svalbard (NIF). We found stronger genetic structure among the two sampled populations in the second reintroduction group (DAU and NIF). Our mtDNA analyses also suggest that introgression possibly occurred from a westward natural recolonization from an unsampled population carrying a haplotype not sampled in other populations, possibly facilitated by more frequent sea ice in the inner parts of the fjord (Muckenhuber et al., 2016). A higher coalescent *N*_e_ in Reintroduction 2 than Reintroduction 1 and the presence of mtDNA haplotypes in both reintroduction groups that were absent from our ADV source population samples is consistent with introgression from an unsampled natural population east of Reintroduction 2. However, these haplotypes may have been present in ADV at the time of reintroduction, but if so it is unclear whether they existed only at low frequencies and increased in reintroduced populations due to founder effects, or if there has been a significant change in the mtDNA haplotype diversity in the ADV population.

### 3.3 Implications for conservation and management

Small or bottlenecked populations are at risk of reduced fitness due to the accumulation of genetic load (i.e. increased frequency and fixation of recessive deleterious mutations), making it an important consideration in conservation biology (Bertorelle et al., 2022; Kardos et al., 2021; van Oosterhout, 2020). Additionally, severe or extended bottlenecks are expected to reduce genome‐wide diversity, including functional genetic variation potentially important for the long‐term adaptive potential of populations (Frankham, 2005; Kardos et al., 2021). Recolonized or reintroduced populations of ibex that have experienced strong founder effects have shown increased realized genetic load compared to those subjected to less severe founder effects (Grossen et al., 2020). Similarly, bottlenecked populations of corvids *Corvus* spp (Kutschera et al., 2020), Montezuma quail *Cyrtonyx montezumae* (Mathur & DeWoody, 2021) and rattlesnakes *Sistrurus* spp (Ochoa & Gibbs, 2021) show higher realized genetic load than larger populations. Thus, the relatively mild founder effects of the Svalbard reindeer reintroductions suggest they are likely to have retained more functional variation and accumulated less realized load than the natural recolonizations, which probably experienced more severe and repeated founder effects. This, in addition to the increased frequency of long runs of homozygosity (that may more strongly reduce fitness) in naturally recolonized populations, means the naturally recolonized, rather than reintroduced populations, likely pose a greater conservation concern. However, while some of the naturally recolonized populations may be at higher risk of reduced fitness due to increased realized genetic load, these populations may also carry fewer highly deleterious recessive mutations due to purging (Glémin, 2003; Grossen et al., 2020), and inbreeding may have less effect on individual fitness (Mathur & DeWoody, 2021).

Several management practice recommendations have been put forward to give general guidelines for the number of individuals that need to be reintroduced, and the amount of gene flow required to maintain genetic diversity. For example, approximately 20 effective founders (Willis & Willis, 2010) and one effective migrant per generation (Vucetich & Waite, 2000) have been viewed as sufficient in mammal populations. Despite founder population sizes lower than this, and the potential for inbreeding depression and accumulation of genetic load, population surveys have shown that both reintroduced and naturally recolonized Svalbard reindeer populations have been expanding (Table S2; Le Moullec et al., 2019), meaning any increase in realized genetic load is not yet severe enough to prevent continued population growth.

Svalbard reindeer face rapid environmental change (Hansen, Pedersen, et al., 2019) that may have implications for the fitness and viability of populations in future. Thus far, most populations of Svalbard reindeer have experienced a net gain from climate change effects, with a warmer and longer snow‐free season leading to increased survival, reproduction and abundances (Albon et al., 2017; Hansen, Pedersen, et al., 2019; Loe et al., 2021). However, an increase in winter precipitation or “rain‐on‐snow” events increases ground ice cover during winter (Peeters et al., 2019) which limits access to winter forage (Hansen et al., 2010; Loe et al., 2016), occasionally destabilising reindeer population dynamics (Kohler & Aanes, 2004; but see Hansen, Gamelon, et al., 2019; Stien et al., 2010). Any short‐ or medium‐term evolutionary responses to such environmental changes will likely depend on sufficient standing genetic variation for natural selection to act upon (Carlson et al., 2014). The potentially genetically depleted naturally recolonized populations may have limited ability to adapt to these changes. There is also the potential for environmental changes to accentuate the fitness effects of realized genetic load. Inbreeding depression (i.e. the fitness effects of realized genetic load) can be more severe in stressful environmental conditions (Armbruster & Reed, 2005; Fox & Reed, 2011). Therefore, previously benign or mildly deleterious variation in reindeer populations may have more severe fitness effects in the future. The Svalbard Archipelago is also experiencing a rapid decline in sea‐ice, which acts as a movement corridor important for facilitating gene flow in Svalbard reindeer (Peeters et al., 2020). Future sea‐ice reductions may further isolate and fragment reindeer populations, so despite the overall population expansion of Svalbard reindeer, accumulation of inbreeding and further loss of diversity in populations subjected to founder effects should remain a concern.

Our results suggest that anthropogenic reintroductions can sometimes be more effective than natural recolonizations in establishing genetically healthy populations, especially under increased anthropogenic landscape fragmentation, such as that due to sea‐ice reductions. This is likely due to a higher number of (potentially more genetically diverse) founders and the avoidance of sequential founder effects that may be an inherent part of the natural recolonization process in fragmented landscapes. These findings also underline that translocations deserve increasing consideration as a tool to augment gene flow required for the adaptation and persistence of populations in fragmented habitats in the context of climate change (Chen et al., 2022) or more generally to maintain diversity and reduce inbreeding (Frankham, 2010). Assisted gene flow could be considered for Svalbard reindeer should populations show signs of decline due to genetic factors in future. Much of the Svalbard archipelago has been recently recolonized by reindeer (i.e. within the last century; Le Moullec et al., 2019), and further sampling may shed more light on whether low diversity and high inbreeding is a general pattern across recolonized populations of Svalbard reindeer, and indeed in recolonized populations of other species that exist in fragmented habitats. Future research may benefit from fitness and phenotypic data, modern and historical reindeer samples, and molecular methods of quantifying both functional variation and genetic load (Bertorelle et al., 2022) to better understand the status of Svalbard reindeer populations and, more generally, how reintroduction and natural recolonization processes affect genetic load, inbreeding depression and potential adaptive variation in the wild.

## 4 MATERIALS AND METHODS

### 4.1 Study area

The Norwegian high‐arctic Svalbard archipelago lies between the Arctic Ocean, the Barents Sea and the Greenland Sea, approximately 700 km north of mainland Norway (76–81° N, 10–35° E). Only 16% of the archipelago's land area comprises vegetated peninsulas and valleys (Johansen et al., 2012), which are fragmented by tide‐water glaciers and inland and mountains that cover the majority of the land area. Vegetation types in the archipelago include polar deserts, Northern Arctic tundra dominated by prostrate dwarf shrubs and cryptogams and Middle Arctic tundra dominated by erect dwarf shrubs, forbs and grasses (Jónsdóttir, 2005).

### 4.2 Study species

The Svalbard reindeer is an endemic subspecies that likely colonized the archipelago from Eurasia 6700 to 5000 years ago (Kvie et al., 2016). The subspecies is the dominant and only large herbivore in the terrestrial ecosystem, with little interspecific competition and almost non‐existent predation pressure (but see Derocher et al., 2000; Stempniewicz et al., 2021). Reindeer were overharvested to near‐extinction on Svalbard during the 19th and early 20th centuries, before coming under legal protection from hunting in 1925 (Le Moullec et al., 2019). By this time, reindeer had been extirpated from much of its former natural range, and isolated remnant populations were largely confined to four regions: the northern, northeastern and eastern extremes of the archipelago, as well as the central Spitsbergen region (Le Moullec et al., 2019; Lønø, 1959). After coming under legal protection, the subspecies began to recover but was still absent from much of its range in the 1970s, including the west coast of Spitsbergen. In 1978, 15 reindeer (with nine females and three males surviving the first months) were translocated from Adventdalen in central Spitsbergen to Brøggerhalvøya on the west coast as part of an ecological experiment (Figure 2; Aanes et al., 2000). The translocation habitat and the source population were chosen due to their proximity to human settlements rather than for the most favourable habitat or genetic factors. In 1984–1985, a second translocation reintroduced 12 individuals to Daudmannsøyra, on the north‐western edge of Isfjorden (Gjertz, 1995), however, there is no population monitoring data to confirm these survived and established the current population. The reindeer population size at Brøggerhalvøya has been annually monitored since the reintroduction (Aanes et al., 2000; Hansen, Pedersen, et al., 2019). This has recorded the population's rapid expansion after translocation (from 12 individuals in 1978 to ~360 individuals in 1993) until a combination of high population density and poor winter conditions triggered a population crash (~80 individuals in 1994) and migration to recolonize the nearby peninsulas of Sarsøyra (1994) and Kaffiøyra (1996) to the south, and Prins Karls Forland island (~1994) to the west (Aanes et al., 2000; Gjertz, 1995).

Reindeer populations have since then recolonized most of their former range naturally, including southern Spitsbergen, the north coast of Isfjorden (to the east of the reintroduced population at Daudmannsøyra), the north‐west coast south to Mitrahalvøya and Wijdefjorden in north‐central Spitsbergen (Le Moullec et al., 2019). Populations at Mitrahalvøya, Wijdefjorden and Southern Spitsbergen appear naturally recolonized from remnant populations, while the origins of the populations along North Isfjorden are unclear, but likely originated from the second reintroduction (Daudmannsøyra) and possibly admixed with naturally recolonizing individuals (Peeters et al., 2020). Both naturally recolonized and reintroduced populations occupy regions with comparable habitat suitability (Pedersen et al., 2023). Genetic evidence suggests the Svalbard reindeer metapopulation has low levels of genetic diversity (Kvie et al., 2016; Weldenegodguad et al., 2020) and shows strong population structure (Côté et al., 2002; Peeters et al., 2020), reflecting a history of population bottlenecks and founder effects and the largely philopatric nature of the species with no large scale migration (Hansen et al., 2010).

### 4.3 Sample collection

Genetic data were generated from tissue samples (ear, antler, bone or fur) collected in 2014–2018 from 100 individual reindeer originating from 12 (sub)populations on the Svalbard archipelago (Figure 2, Tables S3, S4). Based on the extirpation locations reported in Lønø (1959), we categorized these populations as either putative reintroduced or naturally recolonized extirpated populations or remnant non‐extirpated populations. These included six populations believed to have originated from the two translocations (Aanes et al., 2000; Gjertz, 1995): (1) Brøggerhalvøya (BGR, the initial reintroduction site), Sarsøyra (SAR), Kaffiøyra (KAF) and Prins Karls Forland (PKF) from the first translocation (collectively referred to as “Reintroduction 1”) and (2) Daudmannsøyra (DAU, the second reintroduction site) and North Isfjorden (NIF) from the second translocation (hereafter referred to as “Reintroduction 2”). Samples were also collected from the source population of the reintroductions (Adventdalen, ADV), from two other remnant populations (Eastern Svalbard [EST] and North East Land [NE]) and the naturally recolonized populations (Mitrahalvøya [MTR], Southern Spitsbergen [STH] and Wijdefjorden [WDF]). Except for those from Daudmannsøyra (*n* = 8), which are new in this study, all samples were previously used to generate microsatellite data in a study by Peeters et al. (2020).

### 4.4 DNA extraction, library building and sequencing

DNA was extracted from ear tissue for the eight samples from Daudmannsøyra using a Qiagen (Hilden, Germany) DNeasy Blood & Tissue extraction kit according to the manufacturer's instructions except for the addition RNase A (details in SI 1). DNA extraction for all other samples (*n* = 92) is described in Peeters et al. (2020). Genomic library building was performed for all samples based on the method described in (Carøe et al., 2018), and 90 of these were then sequenced to a target depth of 2–3× (see Figure S1, Table S4 for details). These sequencing data were combined with data from deep sequencing of the remaining 10 samples.

### 4.5 Bioinformatic processing and genotype likelihood calculation

We used Paleomix version 1.2.13.4 (Schubert et al., 2014) to map demultiplexed sequence reads to the caribou reference genome assembled from a North American male (Taylor et al., 2019). This reference, while more phylogenetically divergent from the Svalbard reindeer, is more contiguous (*N*_50_ = 11.765 Mbp) than the Mongolian reindeer reference (*N*_50_ = 0.94 Mbp; [Li et al., 2017]) and more suitable for RoH inbreeding‐type analyses. Adapters were trimmed with adapterremoval version 2 (Schubert et al., 2016) and the BWA aligner program version 0.7.15 was used with the MEM algorithm (Li, 2013) without filtering for mapping quality.

To account for the uncertainty in calling genotypes from low‐depth sequencing data, we utilized ANGSD v0.93 (Korneliussen et al., 2014) to generate genotype likelihood data for each individual, and these, rather than explicitly called genotypes, were used in downstream analyses. Genotype likelihood files were generated in beagle format inferring allele frequencies with fixed major and minor alleles using the command‐line arguments *‐doGlf 2* (admixture analyses) or *‐doGlf3* (inbreeding analyses), *‐doMajorMinor 1* and *‐doMaf 1*. Variants were called with a p‐value threshold of 1e^−6^ (*‐SNP_pval 1e‐6*) only at sites for which there was sequence data in at least 50 individuals (*‐minInd 50*). Reads with mapping quality <30 and base quality <20, and those with multiple mapping hits, were filtered out using *‐minMapQ 30*, *‐minQ 20* and *‐uniqueOnly 1*, and low‐quality reads were removed with *‐remove_bads 1*. Scaffolds mapped to bovine sex chromosomes by Taylor et al. (2019) were removed. To reduce any issues related to paralogs or mapping errors we filtered out sites that had average coverage greater than twice or less than ⅓ of the genome‐wide average in a sub‐sample of 10 individuals with equal (3×) coverage. We used the *‐C 50* parameter to adjust map quality for reads with a large number of mismatches to the reference genome, and the extended baq model to adjust quality scores around indels (*‐baq 2*).

#### 4.5.1 Mitochondrial genome analysis

To analyse mtDNA haplotype diversity, we mapped our sequence data to a 16,357 bp reindeer mtDNA reference assembly (Ju et al., 2016). Then, we used the GATK 4.1.8.1 HaplotypeCaller (Depristo et al., 2011) to identify SNPs and call mtDNA haplotypes. Four samples were excluded from the analysis due to low coverage (<20×). We specified haploid calls (*‐ploidy 1*), only used reads with a minimum‐mapping quality of 25 (*‐‐minimum‐mapping‐quality 25*) and specified a confidence threshold of 30 for variant calling (*‐stand‐call‐conf 30*). We then converted the haplotype calls of variable sites to FASTA sequences for each individual. To investigate mitochondrial genetic structure and haplotype diversity, we used pegas v1.1 (Paradis, 2010) to construct a median‐joining haplotype network based on the raw number of nucleotide differences between sequences. We also used pegas to calculate population haplotype richness rarefied to a sample size of five using the Hurlbert (1971) rarefaction method.

#### 4.5.2 Ancestry and admixture analyses

We used the maximum likelihood‐based clustering analysis software package NGSadmix (Skotte & Albrechtsen, 2013) to infer population structure and identify admixture between populations using genotype likelihood data. For admixture analysis, we excluded samples with sequencing depth <0.1× (*n* = 4) and removed two out of three closely related individuals in the reintroduced Daudmannsøyra population identified using NgsRelate (Hanghøj et al., 2019), because closely related individuals can bias admixture results (Garcia‐Erill & Albrechtsen, 2020). We LD pruned the genotype likelihood data by first calling genotypes in ANGSD to generate Tped/Tfam files (*‐doGeno 32* and *‐doPlink 2*) with which we performed variant LD pruning in PLINK v 1.9 (Chang et al., 2015) using *‐‐indep‐pairwise 50 5 0.3* to specify a window size of 50, step size of 5 and a *r* ^2^ threshold of 0.3. Admixture models were run for the number of genetic clusters (*K*) ranging from 2 to 10, with 10 replicates of each. Only sites with a minimum minor allele frequency greater than 0.02 (using *‐minMaf 0.02*) and that had data in at least half (46) of the 92 individuals in the analysis (using *‐minInd 46*) were included in the analysis. We ran admixture analyses on the full dataset including all populations (467,146 sites), and also a separate analysis on a subset including only the reintroduction source, reintroduced and Southern Spitsbergen populations (427,643 sites). For each value of *K*, the replicate with the highest likelihood was selected. We calculated Δ*K* (Evanno et al., 2005) using CLUMPAK (Kopelman et al., 2015), however uneven sampling of ancestral populations and strong genetic drift can bias rule‐based model selection (Garcia‐Erill & Albrechtsen, 2020). Therefore, we considered all *K* models and examined the correlation of residuals using EVALadmix (Garcia‐Erill & Albrechtsen, 2020) to evaluate and interpret results instead of relying solely on a rule‐based model selection procedure.

#### 4.5.3 Principal component analysis

We conducted Principal component analysis (PCA) using the software package PCAngsd (Meisner & Albrechtsen, 2018) to estimate a genetic covariance matrix using individual allele frequencies based on the same genotype likelihood data used in the admixture analysis, then computed eigenvectors and eigenvalues using the *eigen* function in R 3.6 (R Core Team, 2019). To visualize the data, we plotted the first four PC axes, and used ggplot2 (Wickham, 2016) to calculate 95% CI ellipses of the mean principal component coordinates of each natural population and for Reintroduction 1 and 2. We repeated this analysis using only the individuals from the Adventdalen, Southern Spitsbergen and reintroduced populations to characterize fine‐scale population structure.

#### 4.5.4 *F*_ST_ analysis

We quantified population differentiation by estimating pairwise *F*_ST_ between each population using RealSFS in ANGSD v0.93 based on 2D (pairwise) population site frequency spectra (SFS) including all samples. First, we generated unfolded per‐site allele frequencies (SAF) for each population using the *‐dosaf 1* argument in ANGSD with the same parameters and site filtering as when generating genotype likelihoods and specified the reference as the ancestral genome. Then, with the realSFS module in ANGSD, we used the unfolded SAF to generate folded 2D SFS priors for each pair of populations using *‐fold 1* since no ancestral states were available to polarize the ancestral/derived alleles. We then input the unfolded SAFs and the folded 2D SFS prior to realSFS to estimate per‐site and global *F*_ST_, specifying the Hudson estimation method which is more suitable for smaller sample sizes (Bhatia et al., 2013) using ‐‐*whichFST 1*. Finally, we used the realSFS *fst stat* function to calculate the weighted global *F*_ST_ for each population pair.

#### 4.5.5 Heterozygosity

We estimated genome‐wide heterozygosity for each individual with coverage >2.5× using realSFS in ANGSD v0.93 based on the folded site frequency spectrum of each individual (Korneliussen et al., 2014). We used the same site filtering and parameters as for the genotype likelihoods described above, however since coverage can bias heterozygosity estimates in our data (see Figure S11), we downsampled each sample to 2.5× coverage using ‐DownSample in ANGSD to allow unbiased comparisons between the maximum number of samples. To estimate heterozygosity in ANGSD, we generated a folded SFS from unfolded SAF separately for each individual and divided the number of heterozygous sites by the total number of non‐N sites.

#### 4.5.6 Coalescent effective population size

To estimate the coalescent effective population size, we first used ANGSD to calculate the per‐site sample allele frequency (SAF) likelihoods for each population (*‐doSaf 1*) using the same downsampled samples used for the heterozygosity analysis with the same filtering and parameters, but only considering sites with data from a minimum of 2/3 of the population sample size. With the realSFS module in ANGSD, we used the unfolded SAF to generate folded single population SFS, then input the unfolded SAF and folded SFS prior to the realSFS *saf2theta* function to calculate per‐site thetas. We used these per‐site thetas with the thetaStat module *do_stat* function to calculate global Watterson's thetas (Ɵ_W_) for each population. Using the Ɵ_W_ estimates, we estimated the coalescent effective population size based on the equation Ɵ_W_ = 4N_e_μ, assuming a per‐generation mutation rate μ of 3.46 × 10^−8^ as estimated for caribou by Dedato et al. (2022).

#### 4.5.7 Inbreeding and runs of homozygosity

We used ngsF‐HMM (Vieira et al., 2016) to identify tracts of individual genomes identical by descent (hereon referred to as RoH), and estimate inbreeding coefficients from genotype likelihoods. This method utilizes a hidden Markov model approach to estimate per‐site probabilities of being IBD rather than a rule‐based method, and can be used with genotype likelihood data so is more appropriate for use with low‐depth WGS data. We excluded scaffolds shorter than 10 Mbp from inbreeding analyses, leaving approximately 56% of the assembled genome (1.235 Gbp) covered by 1,640,852 variable sites on 64 scaffolds after filtering. We also applied stricter filtering than with heterozygosity, restricting this analysis to samples with >2.5× coverage based on reads used by ANGSD on the >10‐Mbp scaffolds after all filtering parameters, and then downsampled each to 2.7× coverage using samtools (Li et al., 2009) to allow unbiased RoH comparisons between our samples. We inferred the approximate age of inbreeding (i.e. the number of generations back to the common ancestor that an RoH was inherited from) based on RoH lengths, using the equation G = 100/(2rL) where r is the recombination rate and L is the length of RoH in Mbp (Kardos et al., 2017; Thompson, 2013), assuming a recombination rate similar to red deer (*Cervus elaphus*) of ~1 cM/Mbp (Johnston et al., 2017).

## AUTHOR CONTRIBUTIONS

The study was conceived by BBH, HJ, HB, MDM and VCB. MDM, BBH, HJ, LEL and LD provided funding for the study. ÅØP, MLM, BBH and BP collected most samples. HB, VCB, BP and MDM performed laboratory and bioinformatic analyses. HB wrote the manuscript with contributions from all authors.

## CONFLICT OF INTEREST STATEMENT

The authors declare no conflicts of interest.

## Supporting information


Data S1.

Table S4.

## Data Availability

The sequence data generated for this study is available on the European Nucleotide Archive under project accession PRJEB57293. The sample metadata that support the findings of this study are available in the Supplementary Material of this article.
